# Loss of Heterozygosity (LOH) Affecting HLA Genes in Breast Cancer: Clinical Relevance and Therapeutic Opportunities

**DOI:** 10.3390/genes15121542

**Published:** 2024-11-28

**Authors:** María Antonia Garrido, Alba Navarro-Ocón, Víctor Ronco-Díaz, Nicolás Olea, Natalia Aptsiauri

**Affiliations:** 1 Radiology Service, Virgen de la Nieves University Hospital, 18014 Granada, Spain; nona_garrido@hotmail.com (M.A.G.); nolea@ugr.es (N.O.); 2 Department of Biochemistry, Molecular Biology III and Immunology, School of Medicine, University of Granada, 18016 Granada, Spain; albanaoc@correo.ugr.es (A.N.-O.); victor.ronco@genyo.es (V.R.-D.); 3 Biosanitary Research Institute of Granada (ibs.GRANADA), 18012 Granada, Spain; 4 Department of Genomic Medicine, Pfizer-University of Granada-Andalusian Regional Government Centre for Genomics and Oncological Research (GENYO), 18016 Granada, Spain; 5 Department of Radiology and Physical Medicine, School of Medicine, University of Granada, 18016 Granada, Spain; 6 CIBER of Epidemiology and Public Health (CIBERSP), 28034 Madrid, Spain

**Keywords:** HLA class-I, LOH HLA, cancer immune escape, breast cancer

## Abstract

Major histocompatibility complex (MHC) class-I molecules (or Human Leucocyte Antigen class-I) play a key role in adaptive immunity against cancer. They present specific tumor neoantigens to cytotoxic T cells and provoke an antitumor cytotoxic response. The total or partial loss of HLA molecules can inhibit the immune system’s ability to detect and destroy cancer cells. Loss of heterozygosity (LOH) is a common irreversible genetic alteration that occurs in the great majority of human tumors, including breast cancer. LOH at chromosome 6, which involves HLA genes (LOH-HLA), leads to the loss of an HLA haplotype and is linked to cancer progression and a weak response to cancer immunotherapy. Therefore, the loss of genes or an entire chromosomal region which are critical for antigen presentation is of particular importance in the search for novel prognostic and clinical biomarkers in breast cancer. Here, we review the role of LOH-HLA in breast cancer, its contribution to an understanding of cancer immune escape and tumor progression, and discuss how it can be targeted in cancer therapy.

## 1. Introduction

Malignant transformation is accompanied by an accumulation of somatic mutations, genetic instability and chromosomal alterations. Cancer immune escape associated with these aberrations is a serious obstacle in developing effective anticancer therapeutic strategies. Recent advances in the clinical application of newly designed protocols of cancer immunotherapy, including immune checkpoint inhibition and T-cell-mediated therapies, have confirmed that cancer immune evasion mechanisms remain responsible for the resistance to treatment and poor survival [1]. One of these mechanisms is a lack of normal tumor antigen presentation caused by altered HLA class-I (HLA-I) expression on tumor cells. It renders malignant cells invisible to cytotoxic T cells (CTLs) and helps cancers evade immune surveillance and T-cell-based immunotherapies [2]. Loss of heterozygosity (LOH) at different chromosomes is one of the hallmarks of cancer. LOH in 6p21 (LOH-6) and 15q21 (LOH-15), where the *HLA class-I* heavy chain and the light chain (beta2-microglobulin (*B2M*)) genes are located, respectively, represents irreversible defects and is linked to more aggressive clonal tumor evolution and metastatic dissemination [3]. Previous studies have reported a high incidence of HLA-I altered expression in breast cancer (BC), with LOH-HLA as a frequent molecular mechanism causing an HLA haplotype loss [4]. This irreversible HLA-I defect can cause cancer progression and failure of the treatment due to a reduction in the tumor antigenic peptide repertoire that can trigger tumor recognition and rejection. Recently, with more new data coming from pre- and post-immunotherapy tumor studies, LOH-HLA has been recognized as a common mechanism of resistance to immunotherapy. LOH-HLA has been reported in cancer patients who developed a resistant lesion after being treated with immunotherapy using immune checkpoint (ICP) inhibitors or adoptive T-cell transfer [5]. Hence, the detection, characterization and targeting of tumor cells with LOH-HLA is essential for improving cancer patient outcomes. In this review, we highlight the relevance of LOH involving HLA genes and its prognostic value and revise new experimental therapeutic prospects based on targeting tumor cells with LOH-HLA.

## 2. Importance of HLA Class-I Loss in Tumor Rejection and Immune Escape

It is well established that tumor cells evade MHC class-I molecules to avoid recognition and destruction by T lymphocytes [2,6]. This escape route avoids antigen presentation to T cells (tumor peptides) and is frequently detected in solid tumors [7,8]. HLA class-I losses have been reported in bladder [9,10], breast [11,12] cervical [13,14], colorectal [15,16], lung [17,18], melanoma [19,20], pancreas [21,22] prostate [23,24] and thyroid cancers [25], as well as in B-cell lymphomas [26] and glioblastomas [27].

**Figure 1:**
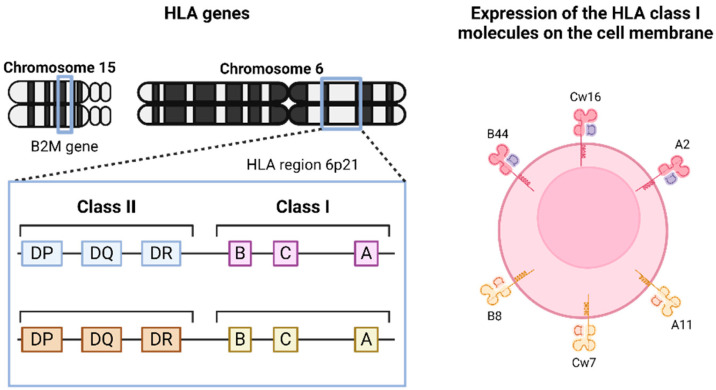
Genomic organization of HLA genes and expression of HLA-I molecules on the cell membrane.

The classical HLA-I complex on the cell surface consists of a heavy alpha chain encoded by three genes (*HLA-A, HLA-B* and *HLA-C*) located on chromosome 6p21, and a light chain encoded by the β2-microglobulin (*B2M*) gene located on chromosome 15q21 [28] (Figure 1).

**Figure 2:**
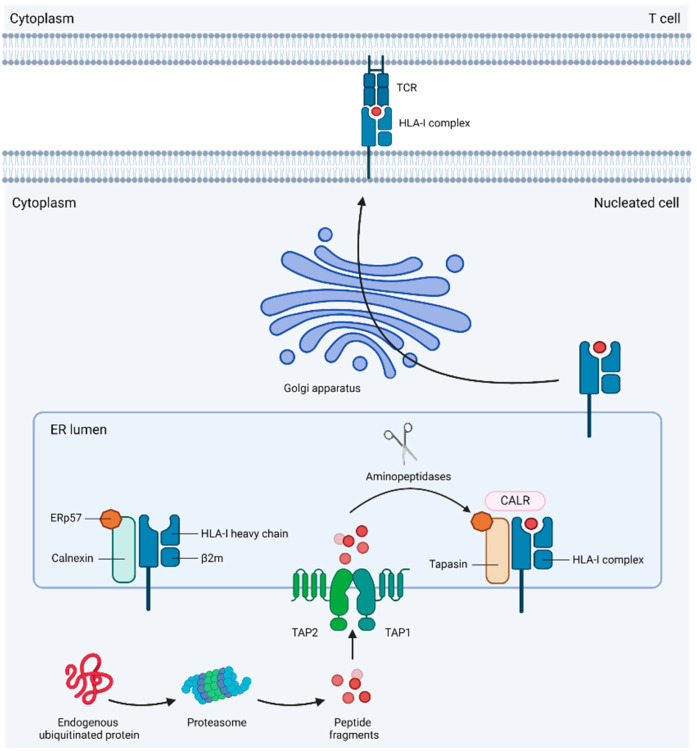
Antigen processing and presentation.

Tumor neoantigens are degraded into peptides and later bound to this complex to stimulate cytotoxic T lymphocytes in a peptide-specific manner. The coordinated activation of various components of antigen processing and presentation machinery (APM) secures successful HLA-I/peptide assembly and transport to the cell surface (Figure 2).

**Figure 3:**
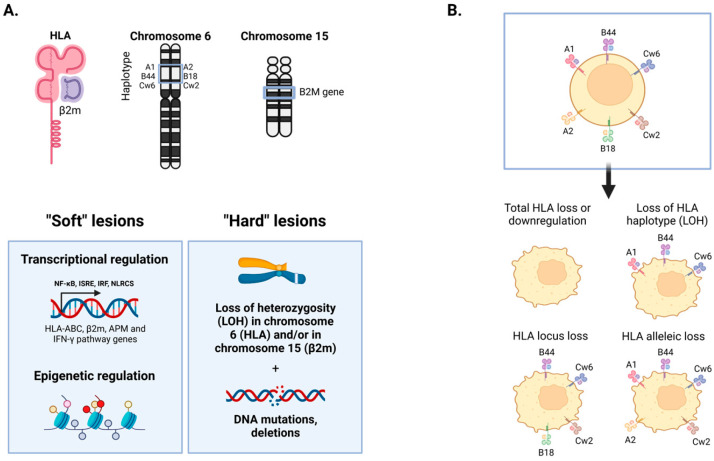
Reversible and irreversible HLA-I alterations (**A**) and corresponding expression profiles on the cell membrane (**B**).

Different molecular mechanisms are responsible for HLA class-I loss, producing different altered HLA class-I tumor phenotypes [2,29], affecting the HLA class-I genes or regulatory pathways that influence the transcription and transduction of HLA molecules. Any step of HLA-I/peptide complex assembly and transport to the cell surface can fail and generate an HLA-I-deficient tumor [30,31]. Years ago, we proposed classifying these mechanisms into reversible (soft) and irreversible (hard) depending on the capacity of tumor cells to recover lost HLA expression by different stimuli [32] (Figure 3). The “hard” mechanisms affect the *HLA* or *B2M* genes due to point mutations or the loss of heterozygosity (LOH) in chromosome 6, where the HLA heavy chain genes are located [33]. The “soft” mechanisms involve the downregulation of HLA genes by transcriptional or epigenetic dysregulation triggered in the tumor microenvironment. For instance, the demethylation of HLA-I genes by 5-azacitidine was reported to induce HLA-I upregulation in melanoma cells [34]. Similarly, in vitro treatment of papillary thyroid cancer cells with interferon and selumetinib can also increase levels of HLA-I [25]. There are some mechanisms that are frequently found in different types of tumors and can be the target of potential therapies. Among those is the loss of heterozygosity (LOH) affecting the HLA genetic region (LOH HLA) at chromosome 6 [35].

## 3. HLA Class-I Alterations and Tumor Immunophenotypes in Breast Cancer

Breast cancer is a result of a multistep process of carcinogenesis, which starts with premalignant lesions and carcinoma in situ followed by invasive carcinoma [36].

This transition is accompanied by tumor infiltration with different types of leukocytes (including myeloid cells), B lymphocytes and cytotoxic CD8 T cells [37,38].

It is well established that breast epithelia and low-risk proliferative lesions of the breast are HLA-I-positive [36,39]. In contrast, in tumor cells, HLA class-I antigens are downregulated in 30–50% of BC cases [11,12,35,40]. An even higher incidence of HLA-I loss in BC was reported (88.5%) when a larger panel of anti-HLA monoclonal antibodies recognizing monomorphic and polymorphic HLA determinants was used, and 39% of these samples demonstrated LOH involving HLA genes [35]. Interestingly, mutations in the B2M gene have not been reported in BC, although the total absence of HLA-I molecules is quite high, mostly due to a coordinated downregulation of HLA and APM molecules or epigenetic alterations along with altered oncogenic and IFN-gamma signaling [41,42,43]. We previously reported that the leading molecular mechanism responsible for HLA-I altered expression in BC is LOH in the HLA-I region of chromosome 6 and in the B2M region of chromosome 15 [35]. BC subtypes had distinct patterns of HLA-I expression; increased LOH-HLA was shown in triple-negative breast cancer [44].

The transcriptional downregulation of HLA-I expression in BC, like in other types of malignancy, is associated with tumor progression and relapse, as well as with lymphatic and nodal invasion [41]. High expression of certain HLA-I molecule subtypes has been linked to an increased 5-year survival rate in BC [45], while HLA-I and APM downregulation is associated with significantly shorter recurrence-free and overall survival [46].

The combination of histology and genomics has provided new molecular subtypes of BC in four groups: luminal A (resembling the histological phenotypes ER+, PR+, HER2− and Ki67−), luminal B (ER+, PR+, HER+/− and Ki67+), HER2 (ER−, PR− and HER2+) and triple-negative subtypes (ER−, PR−, HER2−) [47]. This classification of BC has clinical relevance. Han and co-authors reported that a high HLA-I expression level was generally associated with increased immune cell infiltration during the transition from in situ to invasive, while low expression was associated with poor clinical outcomes in hormone receptor-negative BC, especially in triple-negative subtypes [48]. In addition, the degree and composition of tumor immune infiltration also have prognostic and clinical importance. The four immunophenotypes mentioned above have been characterized by low, intermediate, or high intra-tumoral immune infiltrate. In addition, the morphological distribution of immune cell infiltration is also an important variable in tumor immunophenotype. Thus, “hot” or “inflamed” tumors with an increased presence of CD8+-positive T cells within the tumor mass have a better chance of being eliminated than “cold” tumors with T cells restrained to the tumor-surrounding stroma [49,50]. In addition, “cold” tumors have worse prognosis and poor response to immunotherapy. LOH-HLA has been associated with immune evasion in immunological “cold” tumors in various tumor types [17,51].

Frequently, tumor-infiltrating specific CTLs are exhausted and express high levels of inhibitory immune checkpoint (ICP) molecules, including PD-L1, which bind to their ligands on tumor cells (PD-L1) and inactivate the ability of T cells to reject tumors [52]. Tumors with low or altered HLA-I expression may have increased levels of PD-L1 as an additional mechanism of cancer immune escape that suppresses T-cell activation and cytotoxicity. Tumors with altered HLA-I expression and high PD-L1 upregulation may respond to immune checkpoint blockade therapy, although the lack of proper antigen presentation due to HLA-I loss or downregulation can limit its therapeutic efficacy.

Many factors of the tumor microenvironment, including immunosuppressive cytokines (TGF-beta, IL-10), MDSCs and T-regs, determine the outcome of the antitumor immune response. Another important factor that defines the immunogenicity of cancer and has a significant correlation with HLA-I expression and the ability of CTLs to recognize and kill tumor cells is tumor mutational burden (TMB) [53]. TMB in patients with LOH has been reported to be higher in many types of cancer [54]. BC is not considered a very immunogenic tumor type, as it harbors lower mutational loads than melanoma and lung cancer, but mutational burden varies both within and across breast cancer subtypes [53].

Currently, based on the genomic and immunological profiling of BC, new intrinsic tumor subtypes are being characterized [55]. Using RNA sequencing data from the BC TCGA database, four distinct immune phenotypes were identified based on the expression of immune-relevant genes. These Immunologic Constant of Rejection (ICR) phenotypes, ICR 1–4, define immune-favorable (ICR-4) and immune-unfavorable phenotypes (ICR-1) associated with tumor progression and the survival of BC patients [55]. These parameters and phenotypes influence the antitumoral immune response and efficacy of cancer immunotherapy. Although conventional treatment approaches, such as surgery, radiotherapy, chemotherapy and endocrine therapy, as well as immunotherapy, have become more efficient, BC treatment nevertheless still has limitations, with a high incidence of resistance to therapy. Therefore, in BC subtypes with low T-cell infiltration and presence of HLA-I alterations, reversion of the immune suppression in the TME, stimulation of T-cell migration into the tumor, and the targeting of tumor cells with irreversible HLA-I defects are important future objectives aimed at improving the efficacy of treatment. In this context, the detection and analysis of the mechanism of HLA-I aberrations, including LOH-HLA, become essential, since the incidence of this type of HLA defect in cancer is high. Previously, it had been reported that recurrent bladder tumors after BCG treatment or relapsed tumors in cancer patients treated with ICP inhibitors demonstrate increased post-treatment incidence of LOH-6 and LOH-15 linked to therapy resistance [17,51,56,57,58,59].

## 4. Chromosome Instability and LOH at Chromosome 6 in Breast Cancer and Other Malignancies

Clonal LOH occurs in most types of human tumors and represents an irreversible genetic alteration that distinguishes malignant cells from normal cells. LOH affecting different chromosomes is a hallmark of carcinogenesis. LOH can be caused by total chromosomal loss due to mitotic nondisjunction or by chromosomal deletions due to errors during mitotic recombination and a defective DNA damage response [60]. Inactivation of tumor suppressor genes and immune genes due to the loss of the wild-type allele occurs in many cancers, especially at early stages of neoplastic transformation. It suggests a selection of tumor variants with LOH that favors immune escape during cancer initiation and progression [61]. These events can be simple deletions or can be accompanied by duplications of the remaining allele, giving rise to copy-neutral LOH (CN-LOH) or even to copy-gain LOH events. LOH traditionally can be detected using microsatellite multiplex PCR. However, in the case of a CN-LOH, the karyotype may appear normal, even though no normal genes are present, and it can be detected using array-based profiling and Single-Nucleotide Polymorphism platforms. LOH detection has been used to identify genomic regions that harbor tumor suppressor genes and to characterize different tumor types, pathological stages and progression. Detailed analysis of LOH can help us better understand tumor clonal evolution and the selective pressure mechanisms involving specific genes or pathways.

**Figure 4:**
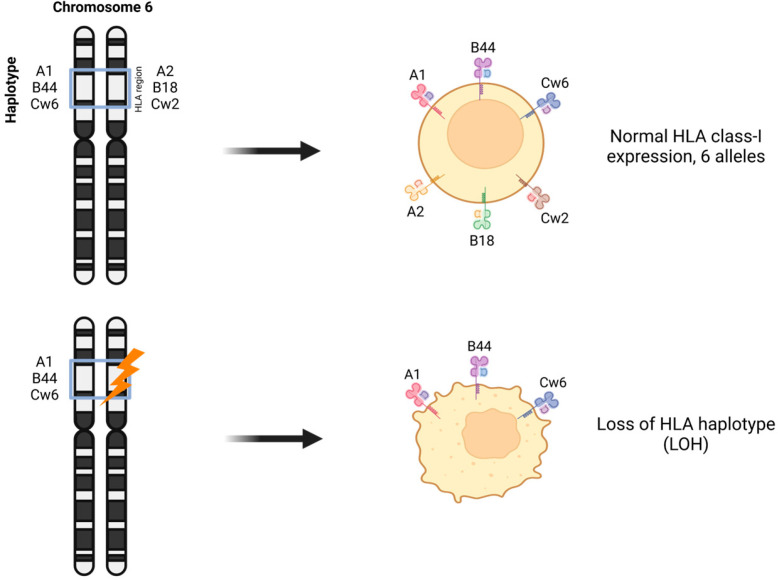
Schematic illustration of HLA-I haplotype loss associated with LOH-HLA.

LOH in chromosome 6 affecting HLA-I heavy chain genes is a frequent mechanism that produces tumor cells expressing one HLA-I haplotype (only three out of six HLA-I alleles) [2,35] (Figure 4). LOH at chromosome region 6p21 occurs in 6–50% of malignancies, depending on the tumor entity; in 36% of laryngeal carcinomas [62]; in 40% of colorectal carcinomas [63]; and in 35% of bladder carcinomas [64]. In BC, we reported LOH at chromosomes 6 and 15, which carry the HLA and B2M genes, in 33% and 25% of cases, respectively [43,65]. More recent publications report a similar incidence of LOH-HLA in non-small-cell lung cancers (40%) [17] and in triple-negative breast tumors (31%) using genomic and computational methods of analysis [66]. Interestingly, in renal cell carcinomas, LOH-HLA has a low frequency [67].

LOH-HLA is a mechanism of immune evasion by tumor cells that results in a decreased repertoire of tumor neo-epitopes presented to T lymphocytes.

LOH in the HLA region might cause a dangerous decrease in HLA allelic diversity and neoantigen binding and is increasingly being recognized as a biomarker of response to cancer immunotherapy. An increasing number of recent publications demonstrate that LOH-HLA plays a role in the clinical and pathological response to treatment [51,54,68]. The response to ICIs is affected by the HLA-I genotype [54]. At the same time, other reports indicate that HLA-I heterozygosity is not associated with the clinical outcome of many cancer patients across eight tumor types treated with pembrolizumab [69]. An association of LOH-HLA with shorter survival has been reported in glioblastoma [27]. LOH-HLA, alone and together with TMB, was reported as a significant negative predictor of overall survival in patients with non-squamous non-small-cell lung cancer [68]. LOH-HLA has been linked to a poor prognosis in adult T-cell leukemia–lymphoma [70]. Demanet and co-authors reported that the selective downregulation of HLA-A and HLA-Bw6, but not HLA-Bw4, is linked to the immune escape of leukemic cells from CTLs and NK cells [71]. Tumors without LOH-HLA show more infiltration by CD8+ T cells. Zang and co-authors have reported that LOH-HLA in lung cancer correlates with other intrinsic and extrinsic biomarkers of carcinogenesis, including an increased driver-gene mutation frequency, oncogenic signaling pathway mutation frequency, tumor mutational burden and chromosomal instability score. Patients with LOH-negative tumors had a longer survival period, and these tumors were more infiltrated by CD8+ T cells [72]. According to Zhou and co-authors, patients with LOH-HLA and a “cold” tumor immunophenotype had a worse prognosis, and breast tumors with LOH-HLA had a higher mutational load [73]. Another report demonstrated that tumors harboring LOH-HLA were more likely to metastasize than those without it [67]. LOH-HLA was observed in early BC and was associated with residual disease [74]. They found evidence for the enrichment of HLA-LOH in lymph node metastasis, suggesting that it may facilitate immune escape and metastatic spread.

LOH-HLA is a “common feature of NSCLC, facilitating immune escape and subclonal genome evolution” [17]. Metastatic spread and tumor clonal progression in BC may also be linked to selective escape by a subclone of tumor cells that has already altered HLA in the primary tumor. Tumors with intrinsic HLA-I may have an advantage in distant organs, causing poor survival. During the process of natural tumor evolution, tumor cells with normal HLA-I expression are eliminated by CTLs, while HLA-I loss variants escape and proliferate.

During this process, the tumor nest undergoes morphological changes in the tumor microenvironment (TME). This includes the gradual loss of HLA-I expression, changes in tumor immune infiltration and the reorganization of stromal elements of the TME. Frequently, at the end of this immune selection process, the tumor becomes “cold”, HLA-negative and is surrounded by a stromal “capsule” enriched with cancer-associated fibroblasts [75].

LOH-HLA could already be present in primary “HLA-I-positive tumors” and undetected if analysis was conducted by immunohistology (IH) using common anti-HLA antibodies. Hence, it is likely that the frequency of LOH-6 and LOH-15 is higher than is estimated. Recent data obtained in our laboratory using SNP GCH arrays and NGS indicate that tumor cells with apparently “normal” HLA expression still harbor HLA gene microdeletions undetected by IH [75]. In addition, we have recently reported that it is possible to detect LOH-6 and LOH-15 in liquid biopsy, in exosomes and cell-free DNA (cfDNA) isolated from tumor cell culture supernatants and the plasma of cancer patients [76].

## 5. Therapeutic Opportunities for Targeting LOH-HLA in Tumor Cells

LOH-HLA is a type of somatic HLA defect that presents an opportunity for personalized targeted therapy in oncology, since it is present only in malignant cells and not in normal ones. The lack of just one HLA-I allele leads to a reduced repertoire of neoantigen presentation and recently has been used to design a therapy using allele-specific gene editing technology to target the remaining HLA allele [77]. T-cell-receptor-restricted therapies and neoantigen vaccines might fail in patients with LOH-HLA, as they may miss alleles responsible for presenting some peptides. Eventually, LOH-HLA analysis will be important not only for patient selection for immunotherapy, but also as a target for a novel therapeutic approach using modified CAR-T cells. The creation of targeted homozygosity in human stem cells has facilitated the generation of specialized cell lines that serve as cellular models for potential CAR-T therapies. It provides standardized cellular platforms that mimic the genetic characteristics observed in patients, allowing for the design of CAR-T cells that specifically target neoantigens or altered antigens resulting from LOH, thereby enhancing the specificity and efficacy of the therapy. Thus, these models facilitate the investigation of the influence of the tumor microenvironment on CAR-T-cell activity as well as the evaluation of treatment efficacy and safety.

Promising CAR-T-cell targets, including CD19, CEA, HER2, MUC1 or Mesothelin, have been identified for the treatment of cancer patients [78,79,80,81,82]. Hwang and co-authors reported a therapeutic approach termed NASCAR (Neoplasm-targeting Allele-Sensing CAR), a platform comprising pairwise chimeric receptors for the detection and targeting of LOH-HLA in cancer [78]. This approach holds significant practical implications by improving the specificity of T-cell therapies, optimizing “on-target, off-tumor” toxicity. NASCAR’s ability to precisely target polymorphic regions lost in cancer cells due to LOH helps distinguish between normal and cancerous tissues, potentially reducing unintended damage and improving treatment safety. Additionally, this approach is adaptable for the targeting of other genetic alterations, such as homozygous deletions or epigenetic changes, further broadening its application across various tumor types. This feature could be crucial for treating refractory cancers that are resistant to conventional therapies. This dual-layer specificity could lead to safer and more effective treatments, especially for solid tumors, and the system could incorporate safety mechanisms, such as suicide gene systems, which enable the controlled elimination of T cells in case of adverse effects.

**Figure 5:**
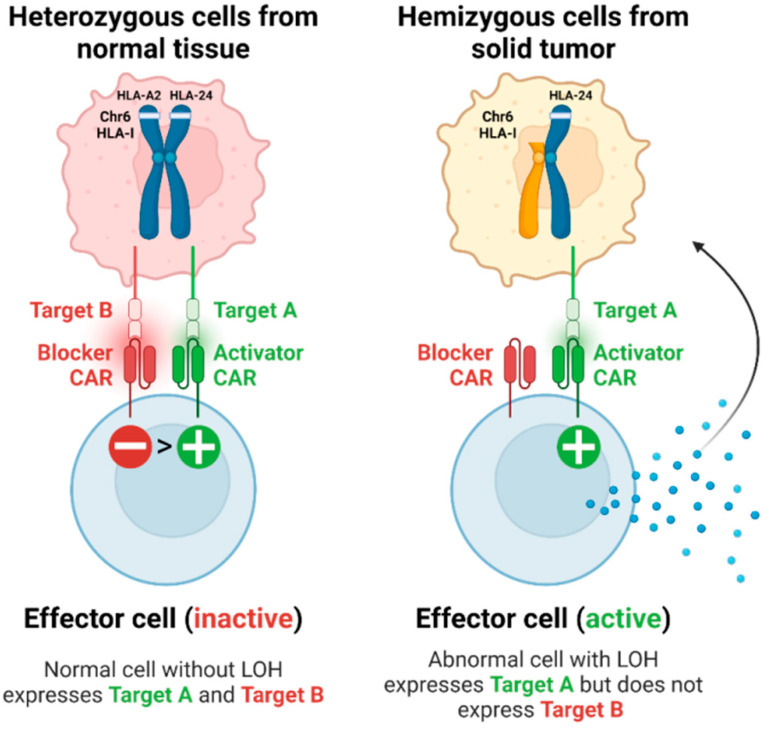
Schematic illustration of the CAR-T cells (with blocker and activator domains) that are activated after recognition of the tumor antigen on a tumor cell that does not express one HLA allele (HLA-A2). Adapted from [78,79].

Hamburger and co-authors used a modular T-cell signal-integration circuit (Tmod2) system to target LOH in solid tumors [79]. It also targets allelic losses common in tumor cells, which enables T cells to selectively attack only cancer cells (Figure 5). The Tmod2 system employs a modular activator/blocker mechanism, allowing it to integrate signals based on the presence or absence of certain antigens. Selective activation ensures that T cells remain inactive against healthy cells but are triggered to attack when they encounter tumor cells exhibiting specific LOH markers. In preclinical studies using mouse models, T cells engineered with Tmod2 demonstrated their ability to selectively kill CD19-positive/HLA-A02-negative tumor cells. The authors demonstrated that the targeting system works both in vitro and in a mouse cancer model where the absence of the HLA-A02 allele releases a brake on engineered T cells activated by the CD19 surface antigen. A similar CAR-T dual-receptor (Tmod™) system with another target antigen, Mesothelin (MSLN), has been described in another publication [80]. By integrating an HLA blocker, the therapy can avoid damaging normal cells expressing the HLA-A2 allele, thereby reducing the risk of side effects such as cytokine release syndrome. Furthermore, the adaptability of the MSLN Tmod construct could allow for its application in patients with various HLA alleles, which broadens its applicability, particularly in regions with specific HLA allele frequencies. Notably, the therapy exploits LOH at the HLA locus, a stable genetic alteration, suggesting that it may retain efficacy even under selective pressures from other treatments, such as checkpoint inhibitors, which can lead to increased rates of HLA loss. The findings also open the door for combination therapies, indicating that MSLN CAR-T therapy could work alongside other treatments targeting different tumor-associated antigens, thereby enhancing overall treatment efficacy.

Another group described the application of CAR-Ts targeting HLA-LOH and Carcinoembriogenic antigen (CEA), which has limited expression in most healthy adult tissues [81]. Here, the activator receptor is also paired with a blocker receptor that recognizes HLA-A2. In tumor cells with HLA-A2 loss, the blocker is no longer activated, allowing the CEA-specific CAR to mediate tumor cell killing. HER-2 has also been successfully used as a target for CAR-Ts that kill tumor cells with a loss of an HLA allele [82].

LOH-HLA generates tumor cells that lose three HLA class-I alleles. However, the above-described CAR-T-mediated experimental therapies target only one missing allele, in this case, HLA-A2, which is more common among the Caucasian population.

In addition, a range of other modalities have been explored to target LOH at the DNA (CRISPR), RNA (antisense oligonucleotide, short hairpin RNA, small interfering RNA), and protein (small-molecule inhibitors) levels. Another approach represents T-cell bispecific antibodies (TCB) bridging CD3ε and HER2 or CEACAM5, which could bypass HLA loss and partially restore T-cell functions [83].

## 6. Conclusions

Loss of heterozygosity (LOH) at chromosome 6, which includes the HLA genomic region, is a frequent finding in BC. Around 35% of breast tumors express only one HLA haplotype (one allele of each locus, *HLA-A*, *-B* and *-C*) drastically reducing the capacity to present potential tumor antigens to T lymphocytes. This tumor escape mechanism described years ago has been recently revisited because of the increasing amount of data demonstrating its correlation with resistance to cancer immunotherapy. There is evidence that the loss of a particular HLA allele is a factor driving tumor evolution and escape during natural cancer progression and in response to therapy. In addition, the association of LOH in the HLA genetic region with tumor progression and poor clinical prognosis suggests a crucial role for these genes in immune selection.

Recent publications describe a new experimental approach targeting tumor cells with LOH in the HLA region using genomic editing or specifically designed CAR-T cells that kill tumor cells that lose only one HLA allele. This approach suggests that the absence of a particular HLA class-I molecule in a tumor cell can be seen not only as a dangerous tumor escape mechanism but also as an opportunity to activate antitumor T cells.

Given the high prevalence of HLA LOH in BC, a greater understanding is needed regarding the impact of this HLA alteration on tumor evolution and response to therapy, and its prognostic value in cancer patients.

## Data Availability

No new data were created or analyzed in this study.

## References

[1] Sharma P., Hu-Lieskovan S., Wargo J.A., Ribas A. (2017). Primary, Adaptive, and Acquired Resistance to Cancer Immunotherapy. Cell.

[2] Garrido F., Ruiz-Cabello F., Cabrera T., Perez-Villar J.J., Lopez-Botet M., Duggan-Keen M., Stern P.L. (1997). Implications for immunosurveillance of altered HLA class I phenotypes in human tumours. Immunol. Today.

[3] Aptsiauri N., Carretero R., Garcia-Lora A., Real L.M., Cabrera T., Garrido F. (2008). Regressing and progressing metastatic lesions: Resistance to immunotherapy is predetermined by irreversible HLA class I antigen alterations. Cancer Immunol. Immunother..

[4] Maleno I., Aptsiauri N., Cabrera T., Gallego A., Paschen A., López-Nevot M.A., Garrido F. (2010). Frequent loss of heterozygosity in the β2-microglobulin region of chromosome 15 in primary human tumors. Immunogenetics.

[5] McGranahan N., Rosenthal R., Hiley C.T., Rowan A.J., Watkins T.B.K., Wilson G.A., Birkbak N.J., Veeriah S., Van Loo P., Herrero J. (2017). Allele-specific HLA loss and immune escape in lung cancer evolution. Cell.

[6] Marincola F.M., Jaffee E.M., Hicklin D.J., Ferrone S. (2000). Escape of human solid tumors from T–cell recognition: Molecular mechanisms and functional significance. Adv. Immunol..

[7] Aptsiauri N., Garrido F. (2022). The Challenges of HLA Class I Loss in Cancer Immunotherapy: Facts and Hopes. Clin. Cancer Res..

[8] Seliger B., Cabrera T., Garrido F., Ferrone F. (2002). HLA class I antigen abnormalities and immune escape by malignant cells. Semin. Cancer Biol..

[9] Flores-Martín J.F., Perea F., Exposito-Ruiz M., Carretero F.J., Rodriguez T., Villamediana M., Ruiz-Cabello F., Garrido F., Cózar-Olmo J.M., Aptsiauri N. (2019). A Combination of Positive Tumor HLA-I and Negative PD-L1 Expression Provides an Immune Rejection Mechanism in Bladder Cancer. Ann. Surg. Oncol..

[10] Cabrera T., Pedrajas G., Cozar J.M., Garrido A., Vicente J., Tallada M., Garrido F. (2003). HLA class-I expresión in bladder carcinomas. Tissue Antigens.

[11] Kaneko K., Ishigami S., Kijima Y., Funasako Y., Hirata M., Okumura H., Shinchi H., Koriyama C., Ueno S., Yoshinaka H. (2011). Clinical implication of HLA class I expression in breast cancer. BMC Cancer.

[12] Giatromanolaki A., Michos G.D., Xanthopoulou E., Koukourakis M.I. (2024). HLA-class-I expression loss, tumor microenvironment and breast cancer prognosis. Cell. Immunol..

[13] Koopman L.A., Corver W.E., van der Slik A.R., Giphart M.J., Fleuren G.J. (2000). Multiple genetic alterations cause frequent and hetero-geneous human histocompatibility leukocyte antigen class I loss in cervical cancer. J. Exp. Med..

[14] Mehta A.M., Jordanova E.S., Kenter G.G., Ferrone S., Fleuren G.J. (2007). Association of antigen processing machinery and HLA class I defects with clinicopathological outcome in cervical carcinoma. Cancer Immunol. Immunother..

[15] Cabrera C.M., Jiménez P., Cabrera T., Esparza C., Ruiz-Cabello F., Garrido F. (2003). Total loss of MHC class I in colorectal tumors can be explained by two molecular pathways: Beta2-microglobulin inactivation in MSI-positive tumors and LMP7/TAP2 downregu-lation in MSI-negative tumors. Tissue Antigens.

[16] Kloor M., Michel S., von Knebel Doeberitz M. (2010). Immune evasion of microsatelite unstable colorectal cancers. Int. J. Cancer.

[17] So T., Takenoyama M., Mizukami M., Ichiki Y., Sugaya M., Hanagiri T., Sugio K., Yasumoto K. (2005). Haplotype loss of HLA class I antigens as an escape mechanism from immune attack in lung cancer. Cancer Res..

[18] Perea F., Bernal M., Sanchez-Palencia A., Carretero J., Torres C., Bayarri C., Gomez-Morales G.F., Ruiz- Cabello F. (2017). The absence of HLA class I expression in non-small cell lung cancer correlates with the tumor tissue structure and the pattern of T cell infiltration. Int. J. Cancer.

[19] Paschen A., Méndez R.M., Jimenez P., Sucker A., Ruiz-Cabello F., Song M., Garrido F., Schadendorf D. (2002). Complete loss of HLA class I antigen expression on melanoma cells: A result of successive mutational events. Int. J. Cancer.

[20] Chang C.-C., Pirozzi G., Wen S.-H., Chung I.-H., Chiu B.-L., Errico S., Luongo M., Lombardi M.L., Ferrone S. (2015). Multiple structural and epigenetic defects in the human leukocyte antigen class I antigen presentation pathway in a recurrent metastatic melanoma following immunotherapy. J. Biol. Chem..

[21] Hiraoka N., Ino Y., Hori S., Yamazaki-Itoh R., Naito C., Shimasaki M., Esaki M., Nara S., Kishi Y., Shimada K. (2020). Expression of classical human leukocyte antigen class I antigens, HLA-E and HLA-G, is adversely prognostic in pancreatic cancer patients. Cancer Sci..

[22] Ryschich E., Notzel T., Hinz U., Autschbach F., Ferguson J., Simon I., Weitz J., Frohlich B., Klar E., Buchler M. (2005). Control of T cell mediated immune response by HLA class I in human pancreatic carcinoma. Clin. Cancer Res..

[23] Seliger B., Stoehr R., Handke D., Mueller A., Ferrone S., Wullich B., Tannapfel A., Hofstaedter F., Hartmann A. (2009). Association of HLA class I antigen abnormalities with disease progression and early recurrence in prostate cancer. Cancer Immunol. Immunother..

[24] Carretero F.J., del Campo A.B., Flores-Martín J.F., Mendez R., García-Lopez C., Cozar J.M., Adams V., Ward S., Cabrera T., Ruiz-Cabello F. (2015). Frequent HLA class I alterations in human prostate cancer: Molecular mechanisms and clinical relevance. Cancer Immunol. Immunother..

[25] Angell T.E., Lechner M.G., Jang J.K., LoPresti J.S., Epstein A.L. (2014). MHC class I loss is a frequent mechanism of immune escape in papillary thyroid cancer that is reversed by interferon and selumetinib treatment in vitro. Clin. Cancer Res..

[26] Challa-Malladi M., Lieu Y.K., Califano O., Holmes A.B., Bhagat G., Murty V.V., Dominguez-Sola D., Pasqualucci L., Dalla-Favera R. (2011). Combined genetic inactivation of β2-microglobulin and CD58 reveals frequent escape from immune recognition in diffuse large B cell lymphoma. Cancer Cell.

[27] Yeung J., Hamilton R., Ohnishi K., Ikeura M., Potter D., Nikiforova M., Ferrone S., Jakacki R., Pollack I., Okada H. (2013). LOH in the HLA class I region at 6p21 is associated with shorter survival in newly diagnosed adult glioblastoma. Clin. Cancer Res..

[28] Beck S., Trowsdale J. (2000). The human major histocompatibility complex: Lessons from the DNA sequence. Annu. Rev. Genom. Hum. Genet..

[29] Garrido F., Algarra I. (2001). MHC antigens and tumor escape from immune surveillance. Adv. Cancer Res..

[30] Jhunjhunwala S., Hammer C., Delamarre L. (2021). Antigen presentation in cancer: Insights into tumour immunogenicity and immune evasion. Nat. Rev. Cancer.

[31] Seliger B., Maeurer M.J., Ferrone S. (2000). Antigen-processing machinery breakdown and tumor growth. Immunol. Today.

[32] Garrido F., Cabrera T., Aptsiauri N. (2010). “Hard” and “Soft” lesions underlying the HLA class I alterations in cancer cells: Implications for immunotherapy. Int. J. Cancer.

[33] Garrido F., Algarra I., García-Lora A.M. (2010). The escape of cancer from T lymphocytes: Immunoselection of MHC class I loss variants harboring structural-irreversible “hard” lesions. Cancer Immunol. Immunother..

[34] Serrano A., Tanzarella S., Lionello I., Mendez R., Traversari C., Ruiz-Cabello F., Garrido F. (2001). Reexpression of HLA class I antigens and restoration of antigen-specific CTL response in melanoma cells following 5-aza-20-deoxycytidine treatment. Int. J. Cancer.

[35] Garrido M.A., Rodriguez T., Zinchenko S., Maleno I., Ruiz-Cabello F., Concha A., Olea N., Garrido F., Aptsiauri N. (2018). HLA class I alterations in breast carcinoma are associated with a high frequency of the loss of heterozygosity at chromosomes 6 and 15. Immunogenetics.

[36] Bombonati A., Sgroi D.C. (2010). The Molecular Pathology of Breast Cancer Progression. J. Pathol..

[37] Salgado R., Denkert C., Demaria S., Sirtaine N., Klauschen F., Pruneri G., Wienert S., Van den Eynden G., Baehner F.L., Penault-Llorca F. (2015). The evaluation of tumor-infiltrating lymphocytes (TILs) in breast cancer: Recommendations by an International TILs Working Group 2014. Ann. Oncol..

[38] Chen Z., Chen X., Zhou E., Chen G., Qian K., Wu X., Miao X., Tang Z. (2014). Intratumoral CD8+ Cytotoxic Lymphocyte Is a Favorable Prognostic Marker in Node-Negative Breast Cancer. PLoS ONE.

[39] Redondo M., García J., Villar E., Rodrigo I., Perea-Milla E., Serrano A., Morell M. (2003). Major histocompatibility complex status in breast carcinogenesis and relationship to apoptosis. Hum. Pathol..

[40] de Kruijf E.M., van Nes J.G., Sajet A., Tummers Q.R., Putter H., Osanto S., Speetjens F.M., Smit V.T., Liefers G.J., van de Velde C.J. (2010). The Predictive Value of HLA Class I Tumor Cell Expression and Presence of Intratumoral Tregs for Chemotherapy in Patients with Early Breast Cancer. Clin. Cancer Res..

[41] Dusenbery A.C., Maniaci J.L., Hillerson N.D., Dill E.A., Bullock T.N., Mills A.M. (2021). MHC Class I Loss in Triple-negative Breast Cancer: A Potential Barrier to PD-1/PD-L1 Checkpoint Inhibitors. Am. J. Surg. Pathol..

[42] Steven A., Seliger B. (2018). The Role of Immune Escape and Immune Cell Infiltration in Breast Cancer. Breast Care.

[43] Critchley-Thornea R.J., Simonsa D.L., Yana N., Miyahiraa A.K., Dirbasc F.M., Johnsonc D.L., Swetterd S.M., Carlsone R.W., Fishere G.A., Koongf A. (2009). Impaired interferon signaling is a common immuneedefect in human cancer. Proc. Natl. Acad. Sci. USA.

[44] Ding X.-H., Xiao Y., Chen F., Liu C.-L., Fu T., Shao Z.-M., Jiang Y.-Z. (2024). The HLA-I landscape confers prognosis and antitumor immunity in breast cancer. Brief. Bioinform..

[45] Stefanovic S., Wirtz R., Sütterlin M., Karic U., Schneeweiss A., Deutsch T.M., Wallwiener M. (2023). Cut-off analysis of HLA-A and HLA-B/C expression as a potential prognosticator of favorable survival in patients with meta-static breast cancer. Anticancer. Res..

[46] Pedersen M.H., Hood B.L., Beck H.C., Conrads T.P., Ditzel H.J., Leth-Larsen R. (2017). Downregulation of antigen presentation-associated pathway proteins is linked to poor outcome in triple-negative breast cancer patient tumors. OncoImmunology.

[47] Hammerl D., Smid M., Timmermans A., Sleijfer S., Martens J., Debets R. (2017). Breast cancer genomics and immuno-oncological markers to guide immune therapies. Semin. Cancer.

[48] Han S.-H., Kim M., Chung Y.R., Woo J.W., Choi H.Y., Park S.Y. (2022). Expression of HLA class I is associated with immune cell infiltration and patient outcome in breast cancer. Sci. Rep..

[49] Bruni D., Angell H.K., Galon J. (2020). The immune contexture and Immunoscore in cancer prognosis and therapeutic efficacy. Nat. Rev. Cancer.

[50] Kirtane K., John M.S., Fuentes-Bayne H., Patel S.P., Mardiros A., Xu H., Ng E.W., Go W.Y., Wong D.J., Sunwoo J.B. (2022). Genomic Immune Evasion: Diagnostic and Therapeutic Opportunities in Head and Neck Squamous Cell Carcinomas. J. Clin. Med..

[51] Chowell D., Yoo S.-K., Valero C., Pastore A., Krishna C., Lee M., Hoen D., Shi H., Kelly D.W., Patel N. (2021). Improved prediction of immune checkpoint blockade efficacy across multiple cancer types. Nat. Biotechnol..

[52] Chow A., Perica K., Klebanoff C.A., Wolchok J.D. (2022). Clinical implications of T cell exhaustion for cancer im-munotherapy. Nat. Rev. Clin. Oncol..

[53] Kandoth C., McLellan M.D., Vandin F., Ye K., Niu B., Lu C., Xie M., Zhang Q., McMichael J.F., Wyczalkowski M.A. (2013). Mutational landscape and significance across 12 major cancer types. Nature.

[54] Shim J.H., Kim H.S., Cha H., Kim S., Kim T.M., Anagnostou V., Choi Y.L., Jung H.A., Sun J.M., Ahn J.S. (2020). HLA-corrected tumor mutation burden and ho-mologous recombination deficiency for the prediction of response to PD-(L)1 blockade in advanced non-small-cell lung cancer patients. Ann. Oncol..

[55] Hendrickx W., Simeone I., Anjum S., Mokrab Y., Bertucci F., Finetti P., Curigliano G., Seliger B., Cerulo L., Tomei S. (2017). Identification of genetic determinants of breast cancer immune phenotypes by inte-grative genome-scale analysis. Oncoimmunology.

[56] Carretero R., Cabrera T., Gil H., Saenz-Lopez P., Maleno I., Aptsiauri N., Cozar J.M., Garrido F. (2010). Bacillus Calmette-Guerin immunotherapy of bladder cancer induces selection of human leukocyte antigen class I-deficient tumor cells. Int. J. Cancer.

[57] Zaretsky J.M., Garcia-Diaz A., Shin D.S., Escuin-Ordinas H., Hugo W., Hu-Lieskovan S., Torrejon D.Y., Abril-Rodriguez G., Sandoval S., Barthly L. (2016). N Mutations Associated with Acquired Resistance to PD-1 Blockade in Melanoma. New Engl. J. Med..

[58] Sade-Feldman M., Jiao Y.J., Chen J.H., Rooney M.S., Barzily-Rokni M., Eliane J.-P., Bjorgaard S.L., Hammond M.R., Vitzthum H., Blackmon S.M. (2017). Resistance to checkpoint blockade therapy through inactivation of antigen presentation. Nat. Commun..

[59] Gettinger S., Choi J., Hastings K., Truini A., Datar I., Sowell R., Wurtz A., Dong W., Cai G., Melnick M.A. (2017). Impaired HLA class I antigen processing and presentation as a mechanism of acquired resistance to immune checkpoint inhibitors in lung cancer. Cancer Discov..

[60] Kumar Y., Yang J., Hu T., Chen L., Xu Z., Xu L., Hu X.-X., Tang G., Wang J.-M., Li Y. (2015). Massive interstitial copy-neutral loss-of-heterozygosity as evidence for cancer being a disease of the DNA-damage response. BMC Med. Genom..

[61] Thiagalingam S., Laken S., Willson J.K.V., Markowitz S.D., Kinzler K.W., Vogelstein B., Lengauer C. (2001). Mechanisms underlying losses of heterozygosity in human colorectal cancers. Proc. Natl. Acad. Sci. USA.

[62] Maleno I., López-Nevot M., Cabrera T., Salinero J., Garrido F. (2002). Multiple mechanisms generate HLA class I altered phenotypes in laryngeal carcinomas: High frequency of HLA haplotype loss associated with loss of heterozygosity in chromosome region 6p21. Cancer Immunol. Immunother..

[63] Maleno I., Cabrera C.M., Cabrera T., Paco L., Lopez-Nevot M.A., Collado A., Ferron A., Garrido F. (2004). Distribution of HLA class I altered phenotypes in colorectal carcinomas: High frequency of HLA haplotype loss associated with loss of heterozygosity in chro-mosome region 6p21. Immunogenetics.

[64] Maleno I., Romero J.M., Cabrera T., Paco L., Aptsiauri N., Cozar J.M., Tallada M., López-Nevot M.A., Garrido F. (2006). LOH at 6p21.3 region and HLA class altered phenotypes in bladder carcinomas. Immunogenetics.

[65] Garrido M.A., Perea F., Vilchez J.R., Rodríguez T., Anderson P., Garrido F., Ruiz-Cabello F., Aptsiauri N. (2021). Copy Neutral LOH Affecting the Entire Chromosome 6 Is a Frequent Mechanism of HLA Class I Alterations in Cancer. Cancers.

[66] Puttick C., Jones T.P., Leung M.M., Galvez-Cancino F., Liu J., Varas-Godoy M., Rowan A., Pich O., Martinez-Ruiz C., Bentham R. (2024). MHC Hammer reveals genetic and non-genetic HLA disruption in cancer evolution. Nat. Genet..

[67] Maleno I., Lopez-Nevot M.A., Seliger B., Garrido F. (2004). Low frequency of HLA haplotype loss associated with loss of heterozygosity in chromosome region 6p21 in clear renal cell carcinomas. Int. J. Cancer.

[68] Montesion M., Murugesan K., Jin D.X., Sharaf R., Sanchez N., Guria A., Minker M., Li G., Fisher V., Sokol E.S. (2021). Somatic HLA Class I Loss Is a Widespread Mechanism of Immune Evasion Which Refines the Use of Tumor Mutational Burden as a Biomarker of Checkpoint Inhibitor Response. Cancer Discov..

[69] Chhibber A., Huang L., Zhang H., Xu J., Cristescu R., Liu X., Mehrotra D.V., Shen J., Shaw P.M., Hellmann M.D. (2022). Germline HLA landscape does not predict efficacy of pembrolizumab monotherapy across solid tumor types. Immunity.

[70] Tamaki T., Karube K., Sakihama S., Tsuruta Y., Awazawa R., Hayashi M., Nakada N., Matsumoto H., Yagi N., Ohshiro K. (2023). A Comprehensive Study of the Immunophenotype and its Clinicopathologic Significance in Adult T-Cell Leuke-mia/Lymphoma. Mod. Pathol..

[71] Demanet C., Mulder A., Deneys V., Worsham M.J., Maes P., Claas F.H., Ferrone S. (2004). Down-regulation of HLA-A and HLA-Bw6 but not HLA-Bw4 allospecificities in leukemic cells: An escape mechanism from CTLs and NK attack. Blood.

[72] Zhang X., Tang H., Luo H., Lu H., Pan C., Yu H., Zhang L., Guan Y., Yu L., Chu H. (2022). Integrated investigation of the prognostic role of HLA LOH in advanced lung cancer patients with immunotherapy. Front. Genet..

[73] Zhou Y.-F., Xiao Y., Jin X., Di G.-H., Jiang Y.-Z., Shao Z.-M. (2021). Integrated analysis reveals prognostic value of HLA-I LOH in triple-negative breast cancer. J. Immunother. Cancer.

[74] Sammut S.J., Crispin-Ortuzar M., Chin S.F., Provenzano E., Bardwell H.A., Ma W., Cope W., Dariush A., Dawson S.J., Abraham J.E. (2022). Multi-omic machine learning predictor of breast cancer therapy response. Nature.

[75] Aptsiauri N., Ruiz-Cabello F., Garrido F. (2018). The transition from HLA-I positive to HLA-I negative primary tumors: The road to escape from T-cell responses. Curr. Opin. Immunol..

[76] Navarro-Ocón A., Cabo-Zabala L., Brinkmann B., López-Sánchez A., Paschen A., Garrido F., Ruiz-Cabello F., Aptsiauri N. (2022). Liquid biopsy for the detection of HLA-I alterations in cancer. HLA.

[77] Nichols C.A., Gibson W.J., Brown M.S., Kosmicki J.A., Busanovich J.P., Wei H., Urbanski L.M., Curimjee N., Berger A.C., Gao G.F. (2020). Loss of heterozygosity of essential genes represents a widespread class of potential cancer vulnerabilities. Nat. Commun..

[78] Hwang M.S., Mog B.J., Douglass J., Pearlman A.H., Hsiue E.H., Paul S., DiNapoli S.R., Konig M.F., Pardoll D.M., Gabelli S.B. (2021). Targeting loss of heterozygosity for cancer-specific immunotherapy. Proc. Natl. Acad. Sci. USA.

[79] Hamburger A.E., DiAndreth B., Cui J., Daris M.E., Munguia M.L., Deshmukh K., Mock J.-Y., Asuelime G.E., Lim E.D., Kreke M.R. (2020). Engineered T cells directed at tumors with defined allelic loss. Mol. Immunol..

[80] Tokatlian T., Asuelime G.E., Mock J.-Y., DiAndreth B., Sharma S., Warshaviak D.T., E Daris M., Bolanos K., Luna B.L., Naradikian M.S. (2022). Mesothelin-specific CAR-T cell therapy that incorporates an HLA-gated safety mechanism selectively kills tumor cells. J. Immunother. Cancer.

[81] Sandberg M.L., Wang X., Martin A.D., Nampe D.P., Gabrelow G.B., Li C.Z., McElvain M.E., Lee W.-H., Shafaattalab S., Martire S. (2022). A carcinoembryonic antigen-specific cell therapy selectively targets tumor cells with HLA loss of heterozygosity in vitro and in vivo. Sci. Transl. Med..

[82] Bassan D., Weinberger L., Yi J., Kim T., Weist M.R., Adams G.B., Foord O., Chaim N., Tabak S., Bujanover N. (2023). HER2 and HLA-A*02 dual CAR-T cells utilize LOH in a NOT logic gate to address on-target off-tumor toxicity. J. Immunother. Cancer.

[83] Messaoudene M., Mourikis T., Michels J., Fu Y., Bonvalet M., Lacroix-Trikki M., Routy B., Fluckiger A., Rusakiewicz S., Roberti M. (2019). T-cell bispecific antibodies in node-positive breast cancer: Novel therapeutic avenue for MHC class I loss variants. Ann. Oncol..

